# Sign change in *c*-axis thermal expansion constant and lattice collapse by Ni substitution in transition-metal zirconide superconductor Co_1−*x*_Ni_*x*_Zr_2_

**DOI:** 10.1038/s41598-023-28291-y

**Published:** 2023-01-18

**Authors:** Yuto Watanabe, Hiroto Arima, Hidetomo Usui, Yoshikazu Mizuguchi

**Affiliations:** 1 Department of Physics, Tokyo Metropolitan University, 1-1, Minami-Osawa, Hachioji, 192-0397 Japan; 2 Department of Physics and Materials Science, Shimane University, Matsue, Shimane 690-8504 Japan

**Keywords:** Condensed-matter physics, Materials for devices

## Abstract

Recently, *c*-axis negative thermal expansion (NTE) was observed in a CoZr_2_ superconductor and related transition-metal zirconides. Here, we investigated the structural, electronic, and superconducting properties of Co_1−*x*_Ni_*x*_Zr_2_ to achieve systematic control of *c*-axis NTE and switching from NTE to positive thermal expansion (PTE) by Ni substitution. At *x* ≤ 0.3, *c*-axis NTE was observed, and the thermal expansion constant *α*_*c*_ approached zero with increasing *x*. At *x* = 0.4–0.6, *c*-axis thermal expansion close to zero thermal expansion (ZTE) was observed, and PTE appeared for *x* ≥ 0.7. On the superconducting properties, we observed bulk superconductivity for *x* ≤ 0.6, and bulk nature of superconductivity is suppressed by Ni heavy doping (*x* ≥ 0.7). For *x* ≤ 0.6, the evolution of the electronic density of states well explains the change in the superconducting transition temperature (*T*_c_), which suggests conventional phonon-mediated superconductivity in the system. By analyzing the *c*/*a* ratio, we observed a possible collapsed transition in the tetragonal lattice at around *x* = 0.6–0.8. The lattice collapse would be the cause of the suppression of superconductivity in Ni-rich Co_1−*x*_Ni_*x*_Zr_2_ and the switching from NTE to PTE.

## Introduction

Thermal expansions are structural properties of materials. In the case of normal (positive) thermal expansion (PTE), an axis and/or volume expand with increasing temperature. In contrast, materials with negative thermal expansion (NTE), those contract with increasing temperature. Importantly, zero thermal expansion (ZTE) can be achieved by fabricating a composite using PTE and NTE materials, and the ZTE materials have been used in various structural materials and devices in which ultraprecision of positions is required^1–5^. However, achievement of ZTE in a single material is quite rare^6^ but has potential merits for development of ZTE application. Development of ZTE in a superconductor is particularly interesting because it will be available in superconducting devices like Josephson junctions with a strength to temperature cycle.

Recently, we reported anomalous axis thermal expansion in CuAl_2_-type (tetragonal) transition-metal zirconide superconductors *Tr*Zr_2_ (*Tr*: transition metal)^7–9^. In CoZr_2_, for example, the *c*-axis shows NTE in a wide temperature range, while the *a*-axis exhibits PTE. Owing to the contrasting axis thermal expansion, CoZr_2_ and similar *Tr*Zr_2_ show volume ZTE in a limited temperature range. In addition, we revealed that the axis ratio *c*/*a* is the potential factor for switching the character of the *c*-axis expansion^9^. In this study, we focus on CoZr_2_ and NiZr_2_ with a large and small *c*/*a* ratio, respectively. CoZr_2_ exhibits a *c*-axis NTE and is a superconductor with a transition temperature (*T*_c_) of ~ 6 K^7,10,11^. NiZr_2_ exhibits PTE in both *a* and *c* axes. In previous works^12–14^, synthesis and physical properties of a solid solution system of Co_1−*x*_Ni_*x*_Zr_2_ were reported with its superconducting properties. Here, we show that the *c*-axis thermal expansion character in Co_1−*x*_Ni_*x*_Zr_2_ is systematically changed from NTE, ZTE, and PTE with increasing Ni concentration *x*.

## Results and discussion

### Crystal structure analysis and axis thermal expansion

**Figure 1:**
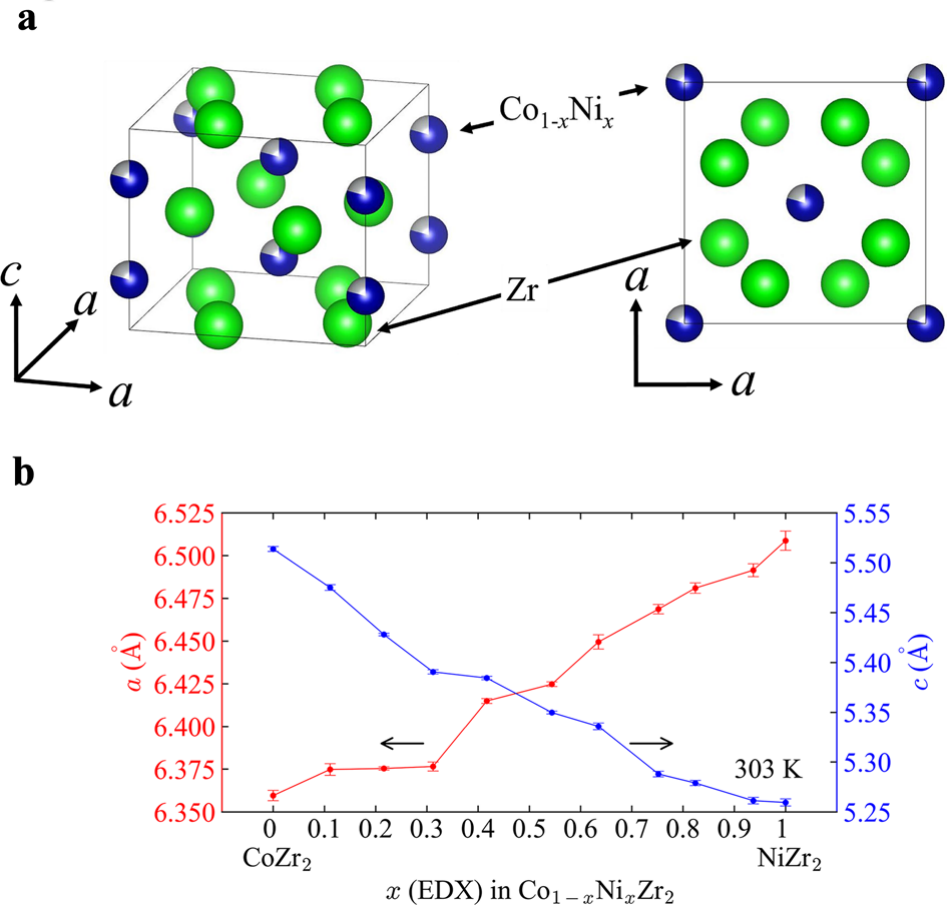
(**a**) Schematic images of the crystal structure of Co_1−*x*_Ni_*x*_Zr_2_. (**b**) Ni concentration (*x*) dependence of lattice constants *a* and *c* at 303 K. The error bars are standard deviations estimated by the Rietveld refinement.

The obtained actual compositions at the *Tr* site are comparable to the nominal values and summarized in Table 1. Figure 1a shows the schematic images of crystal structure of Co_1−*x*_Ni_*x*_Zr_2_. Supplementary Figure S1a (Supplementary data) shows X-ray diffraction (XRD) patterns for *x* = 0–1. These compounds have a tetragonal CuAl_2_-type structure (*I*4/*mcm*), and the main peaks could be indexed with the structural model. The peaks systematically shift by Ni substitution. For example, we clearly see that the 202 and 310 peaks approach each other as *x* is increased. With Ni partial substitutions, changes in peak sharpness and the appearance of multiple sets of peaks cause by CoZr_2_ and NiZr_2_ are not observed. The fact indicates that Ni atoms are uniformly distributed in this sample. Supplementary Figure S1b–d (Supplementary data) show the Rietveld refinement results for *x* = 0, 0.5, 1. Small impurity peaks of the orthorhombic CoZr_3_ phase and/or NiZr phase are seen as reported in Refs.^7,9^. We estimated lattice constants by Rietveld refinements using the XRD patterns at 303 K, and the obtained parameters are plotted in Fig. 1b and summarized in Table 1. Lattice constants consistent with the corresponding XRD peak positions; hence, the influence of the impurity phases is almost negligible in the evaluation of the changes in lattice constants. The obtained trend of lattice constants is consistent with a previous study^14^. The Ni concentrations are obtained using energy-dispersive X-ray spectrometry (EDX).

**Table 1:** Results of chemical analyses, evolutions of lattice constants and thermal expansion constants, and *T*_c_ in examined Co_1−*x*_Ni_*x*_Zr_2_. *T*_c_ with bracket indicates filamentary superconductivity.

Nominal *x*	*x* (EDX)	*a* (Å) at 303 K	*c* (Å) at 303 K	*c/a* at 303 K	*α*_*a*_ (μK^−1^)	*α*_*c*_ (μK^−1^)	*β* (μK^−1^)	*T*_c_ (K)
0	0	6.360 (3)	5.514 (3)	0.8670 (6)	25.6 (6)	− 20 (1)	31 (1)	5.9
0.1	0.111 (3)	6.375 (3)	5.475 (3)	0.8589 (6)	20.2 (8)	− 14 (1)	27 (2)	6.4
0.2	0.216 (6)	6.3750 (9)	5.428 (1)	0.8514 (2)	21.1 (6)	− 4.6 (9)	37 (1)	6.1
0.3	0.312 (2)	6.377 (3)	5.391 (2)	0.8454 (5)	14.9 (6)	− 3 (1)	27 (1)	5.1
0.4	0.417 (3)	6.415 (1)	5.384 (2)	0.8393 (3)	14.0 (7)	− 1 (1)	27 (2)	4.1
0.5	0.544 (6)	6.425 (1)	5.350 (1)	0.8327 (3)	15.1 (5)	1.4 (9)	32 (1)	3.2
0.6	0.635 (5)	6.450 (2)	5.336 (3)	0.8273 (8)	23.6 (7)	2 (2)	48 (2)	2.5
0.7	0.752 (4)	6.469 (3)	5.288 (3)	0.8175 (6)	14.4 (9)	15 (2)	43 (3)	(2.4)
0.8	0.824 (2)	6.481 (3)	5.279 (3)	0.8145 (6)	7 (1)	11 (2)	25 (3)	(2.5)
0.9	0.937 (3)	6.491 (4)	5.261 (3)	0.8105 (7)	10.5 (6)	12 (1)	33 (2)	(2.5)
1	1	6.509 (6)	5.259 (4)	0.8081 (9)	15.8 (5)	17 (1)	49 (1)	(2.0)

**Figure 2:**
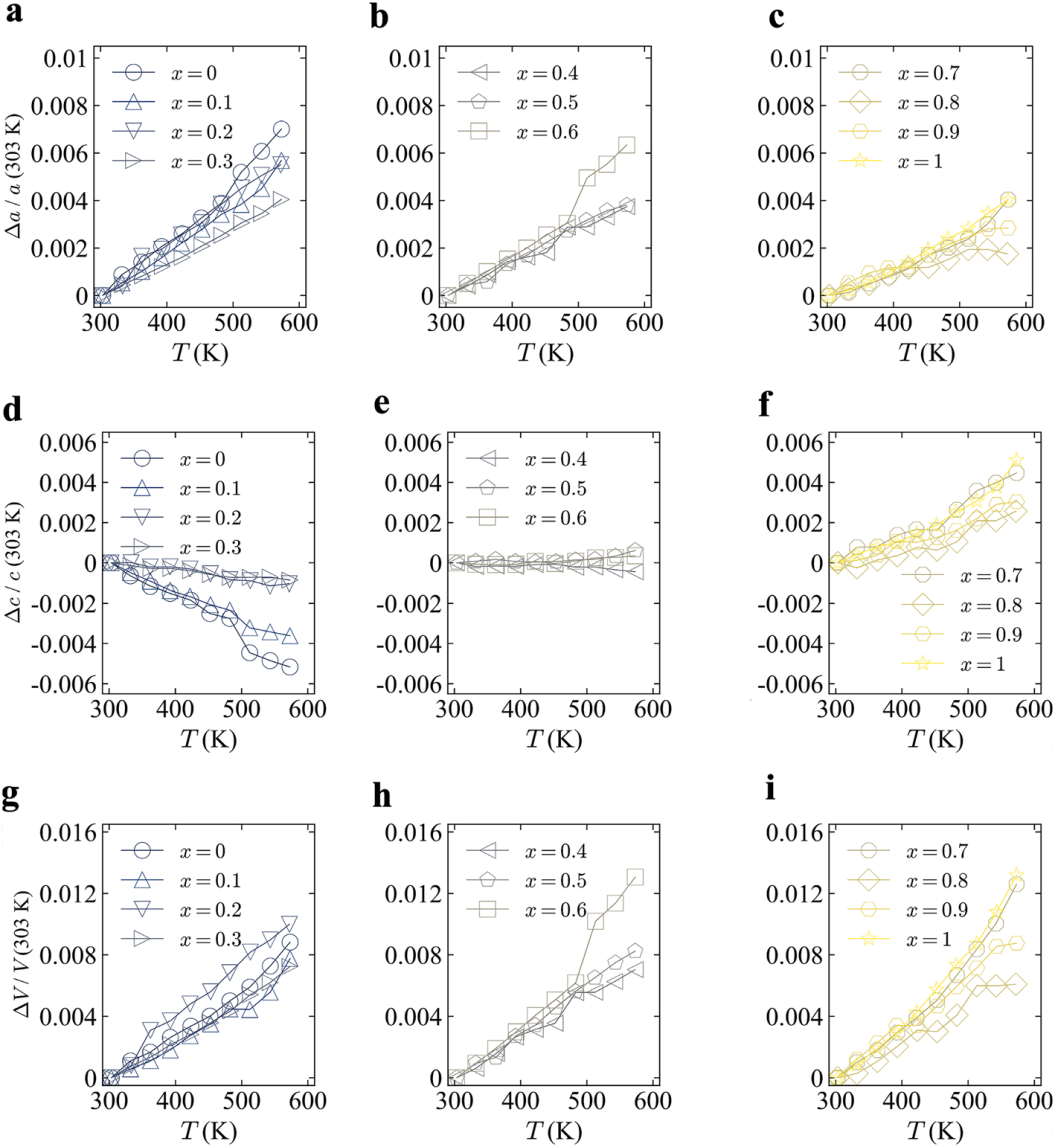
(**a**–**i**) Temperature dependence of the normalized rate of change of the lattice constants *a*, *c*, and *V* from 303 K.

**Figure 3:**
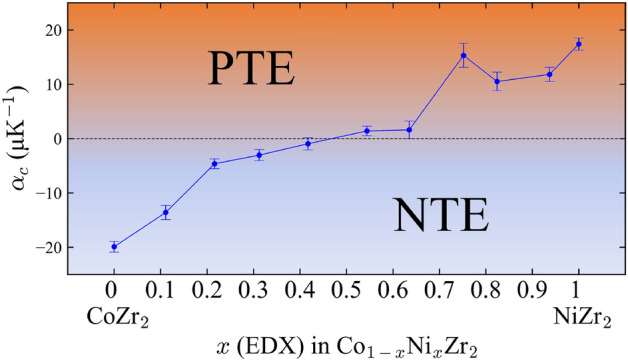
Ni concentration (*x*) dependence of *α*_*c*_ for Co_1−*x*_Ni_*x*_Zr_2_.

We investigated the thermal expansion properties by high-temperature XRD. As reported in Ref. 7, CoZr_2_ exhibits *c*-axis NTE, while the *a*-axis exhibits PTE. To investigate how the anisotropic axis thermal expansion changes with Ni doping, we collected the data for all the samples between 303 and 73 K with a temperature increment of about 30 K and used it for the calculation of the linear thermal expansion coefficients along the *a*-axis (*α*_*a*_) and *c*-axis (*α*_*c*_) and the volumetric expansion coefficient (*β*). The *c*-axis NTE was observed for *x* = 0, 0.1, 0.2, 0.3; hence, we show the results for *x* = 0.3 as an example. Supplementary Figure S2a–c (Supplementary data) show the temperature dependence of lattice constants *a*, *c*, and volume (*V*) for the *x* = 0.3 sample. Supplementary Figure S2d (Supplementary data) shows the typical high-temperature XRD patterns. The 002 peak shifts to the higher angle side with increasing temperature, indicating that the *x* = 0.3 sample still contracts along the *c*-axis upon heating. The estimated values of *α*_*a*_, *α*_*c*_, and *β* using the formulas ${\alpha}_{\text{a}} = \frac{1}{{\text{a}} \, \left(\text{303 K}\right)}{\cdot}\frac{{\text{d}}{\text{a}}}{{\text{d}}{\text{T}}}$, ${\alpha}_{\text{c}} = \frac{1}{{\text{c}} \, \left(\text{303 K}\right)}{\cdot}\frac{{\text{d}}{\text{c}}}{{\text{d}}{\text{T}}}$, and ${\beta} = \frac{1}{{\text{V}}\text{ (303 K)}}{\cdot}\frac{{\text{d}}{\text{V}}}{{\text{d}}{\text{T}}}$ are α_*a*_ =  + 14.9(6), α_*c*_ = − 3(1), *β* =  + 27(1) μK^−1^, respectively The magnitude of α_*c*_ for *x* = 0.3 is smaller than that of CoZr_2_, which also exhibits NTE along the c-axis with *α*_*c*_ < − 15 μK^−1^^7^. This suggests that the substitution of Ni for the Co site suppresses the NTE along the *c*-axis, and the switching between PTE and NTE is controlled by adjusting the *x* value. Figure 2a–i show the temperature dependence of the normalized rate of change in the lattice constants *a*, *c*, and *V* from 303 K for all the samples. For all *x*, the lattice constant *a* and *V* gradually increase with heating. The samples with a lower Ni amount (*x* = 0–0.3) show NTE along the *c*-axis as shown in Fig. 2d. On the other hand, the samples with a larger Ni amount (*x* = 0.7–1) show PTE along the *c*-axis as shown in Fig. 2f. For the samples with medium Ni amount (*x* = 0.4–0.6), ZTE trend was observed as shown in Fig. 2e. Figure 3 shows the *x* dependence of the linear thermal expansion coefficient along the *c*-axis, which shows a successful control of the switching of NTE and PTE along the *c*-axis by tuning *x*. The turning point for the NTE and PTE is estimated between *x* = 0.4 and 0.6. Therefore, there is a possibility to synthesize the sample which exhibits the perfect ZTE along the *c*-axis by optimizing the Ni amount doped at the Co site. As well, materials that exhibit anisotropic thermal expansion have been reported, such as β-Eucryptite (LiAlSiO_4_)^15^, Ag_3_[Co(CN)_6_]^16^, and Ca_2_RuO_4_^17^. The mechanisms of NTE are diverse^1,18^. For example, the cause of the NTE on the monoclinic Ca_2_RuO_4_ is d_*xy*_ orbital ordering and disordering^17^. Not only electronic contributions but also structural properties contribute to the NTE mechanisms. In another study on α-(Cu_2−x_Zn_x_)V_2_O_7_, the chemical substitution of Cu by Zr decreases the free space for the transverse vibrations, which suppresses NTE along the *b*-axis^19^. Furthermore, Mn_3_Cu_1−*x*_Ge_*x*_N exhibits giant negative thermal expansion due to the local lattice distortion triggered by Ge dope^20^. Gao et al. proposed the AAV parameter for discovering the materials which show an isotropic negative thermal expansion^21^. The value of AAV is around 18 Å^3^ for all *x* because their unit cell volume hardly changes with Ni concentration. The AAV may not be essential for the Co_1−*x*_Ni_*x*_Zr_2_ system because they show the anisotropic *c*-axis NTE rather than isotropic NTE; however, parameters common to other anisotropic NTE materials would exist. These facts will help us to understand the mechanisms of the anomalous (anisotropic) *c*-axis thermal expansion in the current system. Recently, we reported that the NTE along the *c*-axis for *Tr*Zr_2_ was caused by the robust *Tr*–Zr distance to the temperature change and the flexible bonding of the *Tr*Zr_8_ polyhedron units. The *Tr*–Zr distance and the Zr–*Tr*–Zr angle are defined in Supplementary Fig. S3a (Supplementary data). Supplementary Figure S3b,c (Supplementary data) show the temperature dependence of the normalized *Tr*–Zr distance and Zr–*Tr*–Zr angle, respectively. The *Tr*–Zr distance seems more robust to temperature change for the lightly Ni-doped samples than the heavily doped samples. The Zr–*Tr*–Zr angle tends to expand with increasing temperature, except for the medium-doped samples. In addition, the *c*/*a* ratio is found to be an essential parameter that determines the polyhedron shape and the thermal expansion characteristics^7,9^. One of the possible reasons for the *c*-axis NTE and the switching from NTE to PTE by Ni substitution would be the difference of bonding state between the Co/Ni and Zr. Since the current investigation has been based on laboratory XRD, we need to perform synchrotron X-ray or neutron diffraction to precisely discuss the Ni substitution effects on crystal structure parameters. Therefore, further studies on electronic and/or orbital characteristics and local structures of Co_1−*x*_Ni_*x*_Zr_2_ will be striking in determination of the mechanisms of the emergence of *c*-axis NTE in *Tr*Zr_2_.

### Superconducting properties

**Figure 4:**
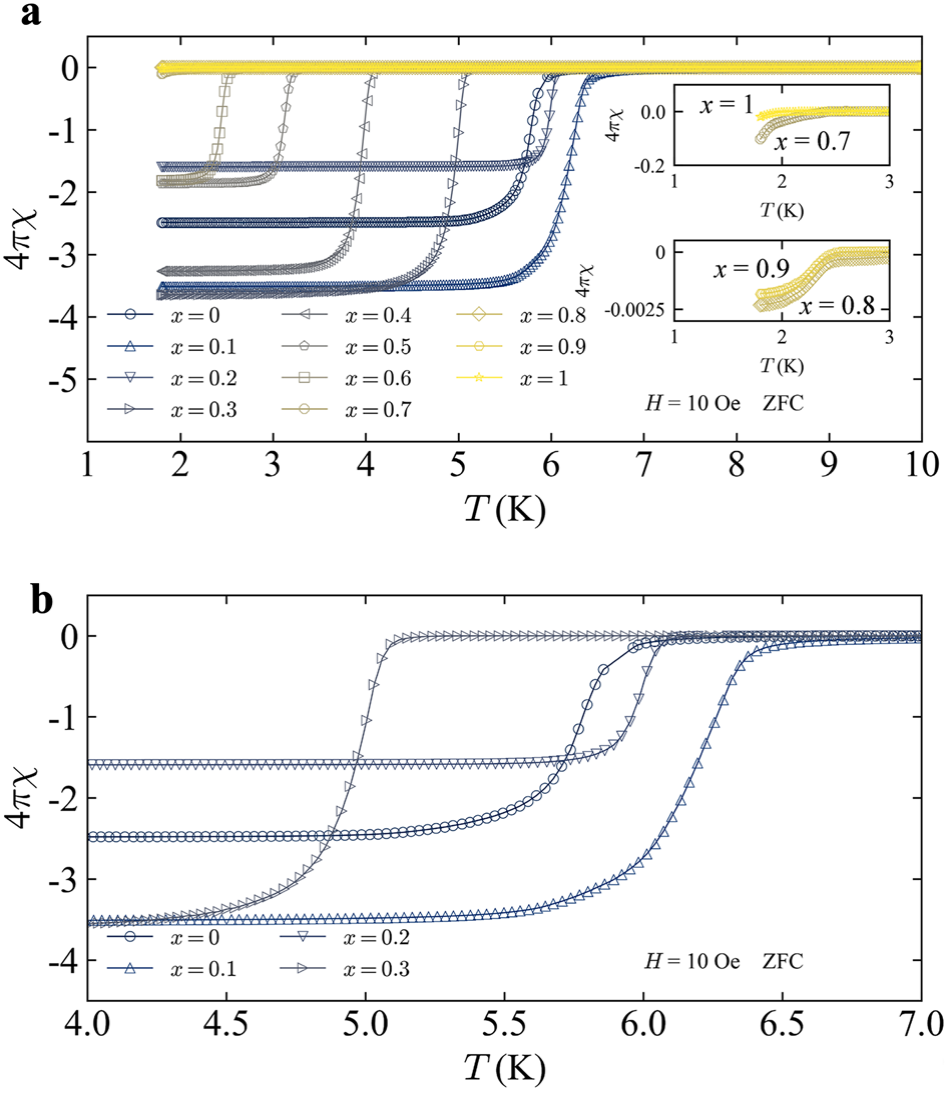
(**a**) Temperature dependence of ZFC susceptibility for Co_1−*x*_Ni_*x*_Zr_2_. (**b**) Enlarged view near the superconducting transition temperature for *x* = 0, 0.1, 0.2, 0.3, 0.4.

**Figure 5:**
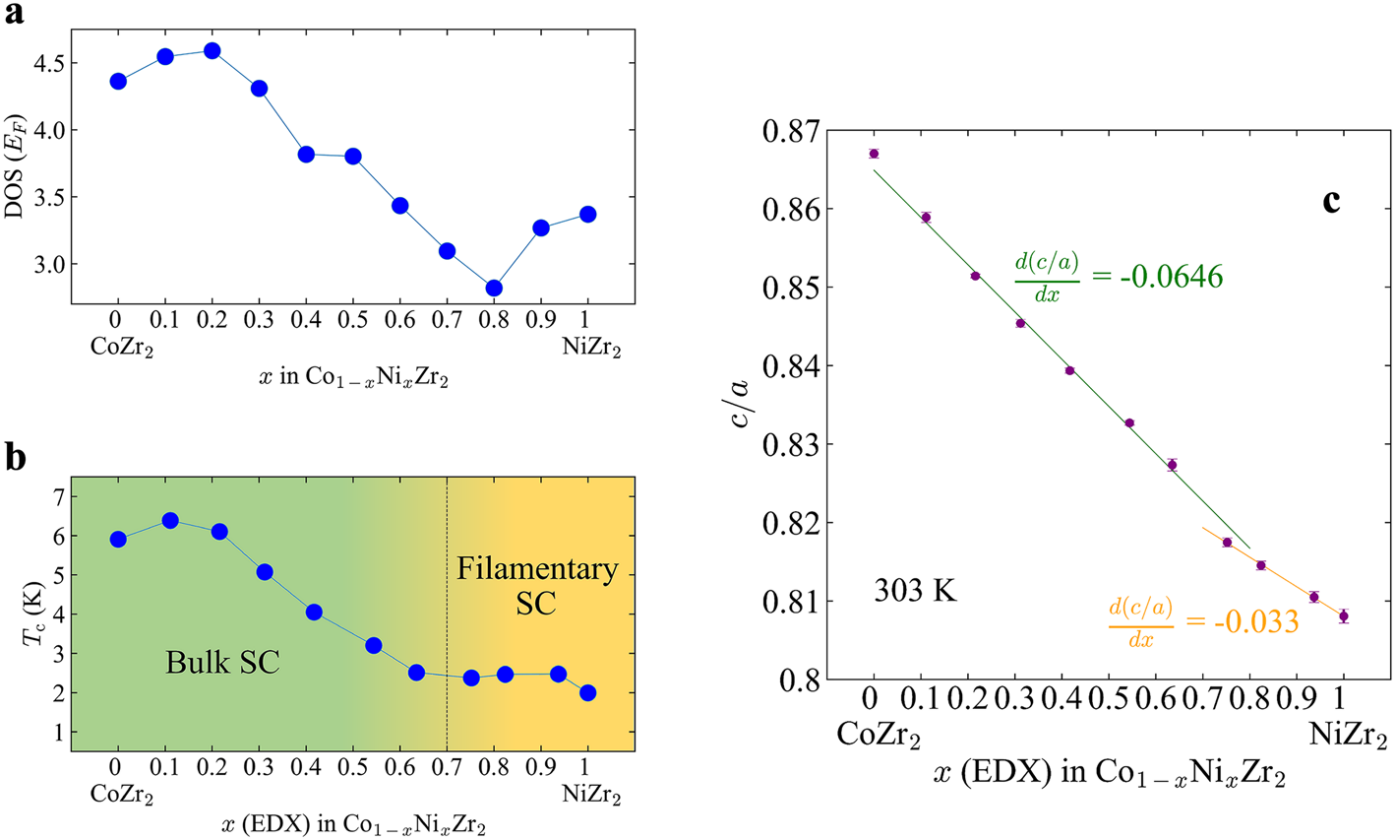
(**a**) The *x* dependence of DOS(*E*_F_) for Co_1−*x*_Ni_*x*_Zr_2_. (**b**) The *x* dependence of superconducting transition temperature for Co_1−*x*_Ni_*x*_Zr_2_. (**c**) The *x* dependence of *c/a* for Co_1−*x*_Ni_*x*_Zr_2_ at 303 K. The estimated slope values for *x* = 0–0.6 and 0.7–1 are − 0.0646 ± 0.0009, − 0.033 ± 0.003 respectively.

A superconducting transition was observed for all Co_1−*x*_Ni_*x*_Zr_2_ samples, as shown in Fig. 4a. Both CoZr_2_ and NiZr_2_ are known to be a superconductor^12–14^ with *T*_c_ of ~ 5 K and 1.6 K, respectively^14^. In addition, it has been reported that *T*_c_ increases with slight Ni substitution in Co_1−*x*_Ni_*x*_Zr_2_ due to the enhancement of spin fluctuation^12^. However, for Ni-rich samples, detailed investigation on superconducting properties, particularly bulk nature of the superconductivity, has not been reported. Our results are basically consistent with previous works. There is no multiple superconducting transition for all samples between 1.8 and 10 K, which is another proof of homogeneous (systematic) Ni substitution in Co_1−*x*_Ni_*x*_Zr_2_. The saturated susceptibility values at the lowest temperature are somehow scattered due to diamagnetic effect, which exhibits the sample-shape dependence on susceptibility^22^. However, the large diamagnetic signals observed for *x* ≤ 0.6 suggests the emergence of bulk superconductivity. In contrast, the signals for *x* > 0.6 are clearly small as a bulk superconductor, which indicates that the observed diamagnetic signals are caused by filamentary (trace) superconductivity states in those samples. In superconductors, whose superconducting states are emerging in the vicinity of magnetic ordering (or strong spin fluctuations), similar filamentary superconductivity has been observed as doped iron-based superconductors (particularly with a collapsed tetragonal structure) are^23,24^. The *T*_c_ tends to decrease with increasing *x*; however, the samples with *x* = 0.1 and 0.2 have a *T*_c_ slightly higher than that for *x* = 0. Figure 4b shows the enlarged view near the *T*_c_ for = 0, 0.1, 0.2, 0.3, 0.4. The highest *T*_c_ of 6.39 K was observed for *x* = 0.1. To discuss about the electronic origins on this behavior, we performed first-principles calculations for Co_1−*x*_Ni_*x*_Zr_2_. Figure 5a shows the *x* dependence of density of states near Fermi level, DOS(*E*_F_). The *x* dependence of the calculated DOS(*E*_F_) looks consistent with the evolution of *T*_c_ if we assumed conventional phonon-mediated superconductivity^25^, because a large DOS(*E*_F_) achieves a higher *T*_c_ in a conventional superconductor. However, we consider possible scenario where phonon and spin-fluctuation scenarios are collaborating in the superconductivity. Takekuni et al. proposed that spin density fluctuations are more essential to superconductivity of Co_1−*x*_Ni_*x*_Zr_2_ rather than DOS(*E*_F_) because there is an enhancement of the nuclear spin–lattice relaxation rate at low temperature in *x* = 0.1^12^. Although our calculation results on DOS(*E*_F_) are consistent with phonon-mediated pairing scenario, other mechanisms with spin fluctuations would be collaborating on the superconductivity. Figure 5b shows the *x* dependence of *T*_c_. As we mentioned above, the evolution of *T*_c_ at *x* = 0.1 is consistent to the DOS(*E*_F_) behavior where the *x* value is smaller than *x* = 0.7. However, we cannot explain the change in *T*_c_ with DOS(*E*_F_) behavior where *x* is larger than *x* = 0.7. As mentioned above, the samples with *x* ≥ 0.7 exhibit filamentary superconductivity; in Fig. 5b, we indicated the boundary between bulk superconductivity (Bulk SC) and filamentary superconductivity (Filamentary SC).

For *x* ≥ 0.7, bulk superconductivity is suppressed, while the DOS(*E*_F_) is comparable or higher than *x* = 0.6. To explore possible cause of the suppression of superconductivity, we estimated the *c*/*a* ratio of Co_1−*x*_Ni_*x*_Zr_2_ using the data at 303 K and plotted in Fig. 5c as a function of *x*. Although *c*/*a* linearly decreases with increasing *x* for *x* ≤ 0.7, the slope clearly changes at around *x* = 0.6–0.8. For *x* = 0.7–1, another slope can guide the evolution of *c*/*a*. This change in the *c/a* slope would be maintained even at low temperatures because the variation of the normalized rate of the change in lattice constants to temperature is not so large as shown in Fig. 2. In fact, axes thermal expansion of CoZr_2_ are monotonic down to 10 K^7^. We propose that the change in the *c*/*a* ratio as shown in Fig. 5c is a kind of transition to collapsed tetragonal phases as observed in iron-based superconductors CaFe_2_As_2_ and KFe_2_As_2_ and related layered compound^26–30^. The electronic structure is generally affected by a collapsed tetragonal transition, which affects superconductivity as well^31,32^, we assume that the disappearance of bulk superconductivity by Ni heavy doping is related to the collapsed transition. In our previous work, we suggested the trend that a higher *T*_c_ of *Tr*Zr_2_ is achieved with increasing lattice constant *c*^33^. This fact is also consistent with the above scenario because the *c*-axis is largely compressed at around *x* = 0.7. To obtain further evidence on the collapsed transition and its relation to electronic structure, superconductivity, and axis thermal expansion, further investigations with different probes are needed. Teruya et al. reported superconducting and Fermi surface of single crystal CoZr_2_^11^. Investigation on physical properties on single crystals of Ni-doped CoZr_2_ will enrich the understanding of superconducting properties and axes thermal expansion in the system.

## Conclusion

We investigated the crystal structure, axis thermal expansion, electronic structure, and superconducting properties of transition-metal zirconide superconductor Co_1−*x*_Ni_*x*_Zr_2_. The samples were synthesized by arc melting and characterized by powder XRD and EDX. At *x* ≤ 0.3, *c*-axis NTE was observed, and the thermal expansion constant (*α*_*c*_) approached zero with increasing *x*. At *x* = 0.4–0.6, *c*-axis thermal expansion close to ZTE was observed, and PTE appeared for *x* ≥ 0.7. Those results confirm that the *c*-axis NTE can be controlled by Ni substitution (tuning *c*/*a* ratio) and switched to PTE. On the superconducting properties, we observed bulk superconductivity for *x* ≤ 0.6, and bulk nature of superconductivity is suppressed by Ni heavy doping. For *x* ≤ 0.6, the evolution of the electronic DOS(*E*_F_) well explains the change in *T*_c_, but it cannot explain the disappearance of bulk superconductivity at *x* ≥ 0.7. By analyzing the *c*/*a* ratio, we revealed a possible transition to collapsed tetragonal phases with a boundary concentration of *x* = 0.6–0.8 by Ni heavy doping. The lattice collapse would affect electronic structure and be negatively linked to superconductivity in Co_1−*x*_Ni_*x*_Zr_2_. In addition, the lattice collapse seems to be linked to the appearance of *c*-axis PTE. Since superconductivity in Co_1−*x*_Ni_*x*_Zr_2_ would be mediated by phonon, the correlation between axis thermal expansion, emergence of superconductivity, and the lattice collapse transition is one of the notable features of this system. Thus, Co_1−*x*_Ni_*x*_Zr_2_ is a suitable platform to study anomalous axis thermal expansion and the method to systematically control the thermal expansion. Furthermore, the relationship between lattice collapse and/or anomalous axis thermal expansion and emergence of superconductivity would provide us with new strategy on exploration of new superconductors.

## Methods

Polycrystalline samples of Co_1−*x*_Ni_*x*_Zr_2_ (*x* = 0, 0.1, 0.2, 0.3, 0.4, 0.5, 0.6, 0.7, 0.8, 0.9, 1) were synthesized by arc melting in an Ar atmosphere. Powders of pure transition metals (*Tr*) of Co (99%, Kojundo Kagaku) and Ni (99.9%, Kojundo Kagaku) with a nominal composition were mixed and pelletized. The *Tr* pellet and plates of pure Zr (99.2%, Nilaco) were used as starting materials. The samples were melted five times and turned over after melting to homogenize the sample.

X-ray diffraction (XRD) patterns were collected by *θ*-2*θ* method with Cu-Kα radiation on a Miniflex-600 (RIGAKU) diffractometer equipped with a high-resolution semiconductor detector D/tex-Ultra. For High-temperature XRD on a Miniflex-600, the sample temperature was controlled by a BTS 500 attachment. The obtained XRD patterns were refined by the Rietveld method using RIETAN-FP^34^, and the schematic images of the crystal structure were depicted using VESTA^35^. The actual compositions of the samples were investigated using energy-dispersive X-ray spectrometry (EDX, Swift-ED, Oxford) on a scanning electron microscope (SEM, TM3030, Hitachi Hightech). We measured randomly-selected ten points on the sample surface, and the actual content of Ni relative to Co is given by the mean value with standard errors. The temperature dependence of magnetization was measured both after zero-field cooling (ZFC) and field cooling (FC) using a superconducting quantum interference device (SQUID) on an MPMS3 (Quantum Design).

The first principles band calculations were performed using the WIEN2k package^36^ within the PBE-GGA exchange–correlation functional^37^. The virtual crystal approximation is adopted to take into account the effect of the elemental substitution of Ni for Co. We used the experimentally determined lattice parameters shown in Table 1. The atomic coordinates of Zr were theoretically optimized. *RK*_max_ and the *k*-mesh were set to 8 and 10 × 10 × 10, respectively.

## Supplementary Information

Supplementary Information.

## Data Availability

All data can be provided by a reasonable request to corresponding author (mizugu@tmu.ac.jp).
