# Supplementary material for: Sign change in c-axis thermal expansion constant and lattice collapse by Ni substitution in transition-metal zirconide superconductor Co1−xNixZr2
Source: Sci Rep. 2023 Jan 18;13:1008. doi: 10.1038/s41598-023-28291-y (PMC9849259; doi:10.1038/s41598-023-28291-y)
Supplement: Supplementary file 1 — Supplementary Information. [file 41598_2023_28291_MOESM1_ESM.pdf]

Supplementary material

Sign change in *c*-axis thermal expansion constant and lattice collapse by Ni substitution in transition-metal zirconide superconductor $Co_{1-x}Ni_xZr_2$

Yuto Watanabe[1], Hiroto Arima[1], Hidetomo Usui[2], Yoshikazu Mizuguchi[1]*

1. Department of Physics, Tokyo Metropolitan University, 1-1, Minami-osawa, Hachioji 192-0397, Japan.
2. Department of Physics and Materials Science, Shimane University, Matsue, Shimane 690-8504, Japan

S.1 The XRD patterns for x = 0−1.

We measured XRD patterns for all samples (See Fig. S1 (a)). Major peaks could be indexed with a tetragonal $CuAl_2$-type structure (*I*4/*mcm*, space group #140); however, some impurity peaks were observed as shown in Fig. S1 (b)-(d). These impurity peaks are originating from an orthorhombic $CoZr_3$ (*Cmcm*, space group #63) phase and/or NiZr (*Cmcm*, space group #63) phase [S1, S2]. We included those impurity phases in the refinements.

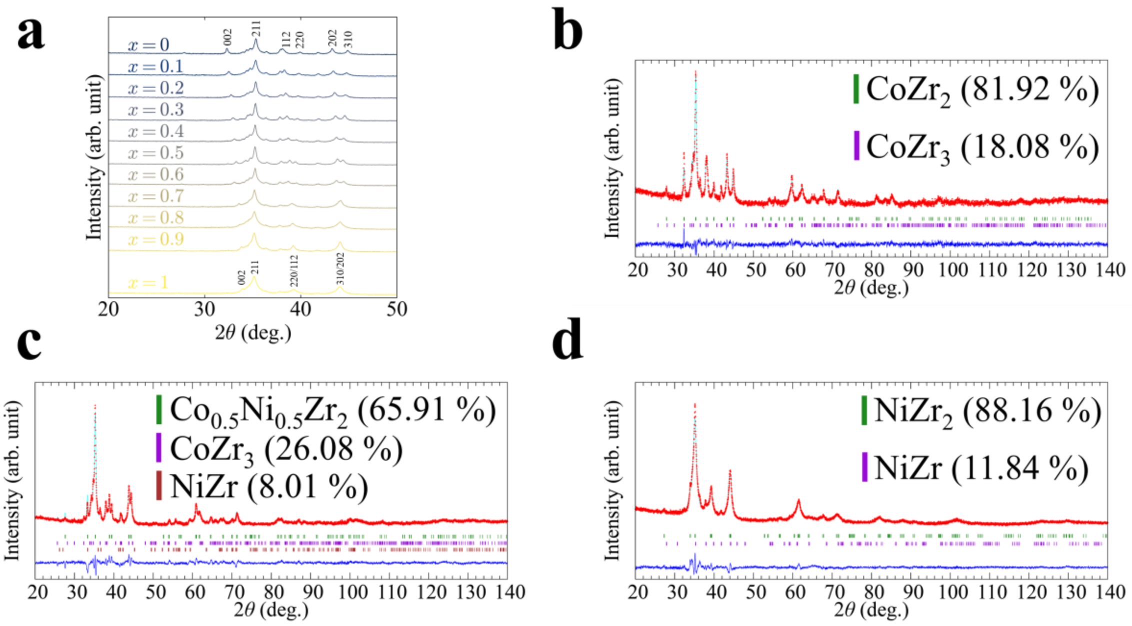

Figure S1. (a) XRD patterns of $Co_{1-x}Ni_xZr_2$ for x = 0–1.0. The numbers are Miller indices. **(b–d)** Rietveld refinement results for **(b)** x = 0, **(c)** x = 0.5, and **(d)** x = 1.

S.2 Thermal expansion results for x = 0.3

We observed the thermal expansion for all samples; and we show the typical results of x = 0.3, which is a boundary between NTE and ZTE. The errors are large as compared to the changes in c, but the analyses resolution in this study can detect the axis thermal expansion for samples near the ZTE composition. This sample exhibits a-axis and volume PTE and c-axis NTE as shown in Supplementary Fig. 2(a)-2(c). Supplementary Figure 2(d) shows high-temperature XRD patterns and the gradual shift of the 002 peak to the higher angle side. This behavior is consistent with the result of c-axis lattice contract.

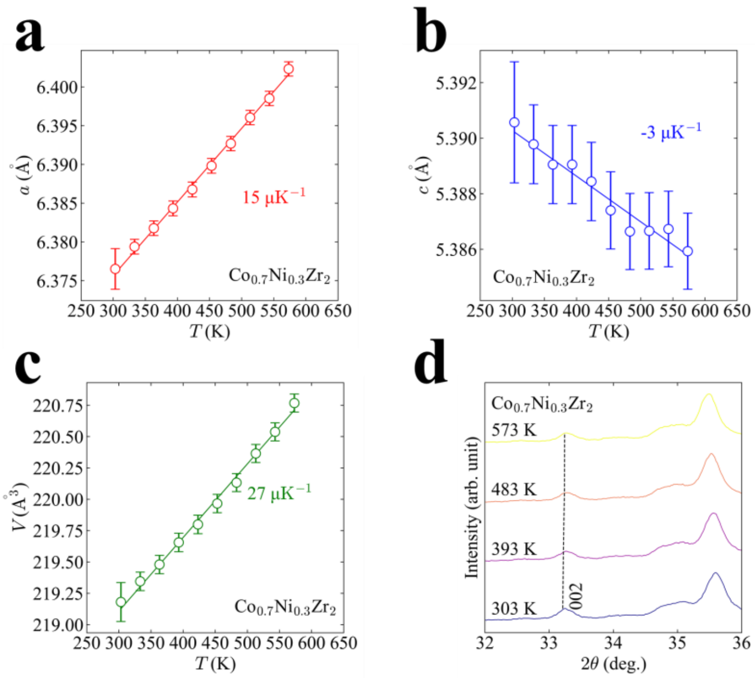

Figure S2. (a, b, c) The temperature dependence of lattice constants a, c, and V for x = 0.3 sample. The estimated linear thermal expansion constants are α_a = +14.9(6) and α_c = -3(1) μK^{-1}. The volumetric expansion coefficient is β = +27(1) μK^{-1} **(d)** High-temperature XRD patterns on T = 303, 393, 483, 573 K for x = 0.3 sample. The dashed line in this graph indicates that the 002 peak shifts to the higher angle side.

S.3 The temperature dependence of the *Tr*-Zr distance and the Zr-*Tr*-Zr angle

The *Tr*-Zr distance and the Zr-*Tr*-Zr angle are defined in Fig. 3(a). We estimated those parameters by Rietveld refinement at each temperature. Since those data are based on laboratory XRD, synchrotron x-ray or neutron diffraction is needed to reliably evaluate structural parameters in future work. The temperature dependence of the *Tr*-Zr distance and the Zr-*Tr*-Zr angle are shown in Fig. 3(b) and Fig. 3(c) respectively. The *Tr*-Zr distance tends to be more robust to temperature change for the lightly Ni-doped samples (for instance, x = 0–0.2) than the heavily doped samples (for instance, x = 0.7–1), and the Zr-*Tr*-Zr angle tends to expand with increasing temperature except for the medium-doped samples (for instance, x = 0.4, 0.5). Those features are consistent with a previous report [S1] for the compositions with NTE.

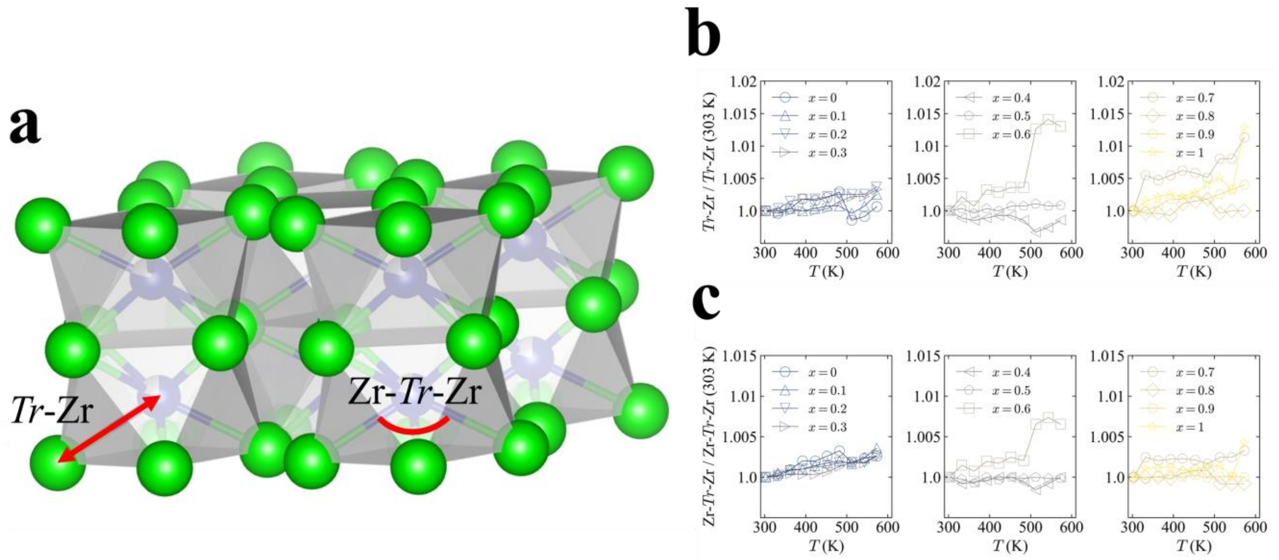

Figure S3. (a) The crystal structure for $Co_{1-x}Ni_xZr_2$ and $TrZr_8$ polyhedrons. The *Tr*-Zr distance and the Zr-*Tr*-Zr angle are depicted. The temperature dependence of **(b)** the *Tr*-Zr distance and **(c)** the Zr-*Tr*-Zr angle.

References

S1) Mizuguchi, Y., Kasem, M. R. & Ikeda, Y. Anomalous Thermal Expansion in a $CuAl_2$-type Superconductor $CoZr_2$. *J. Phys. Soc. Jpn.* **91**, 103601 (2022).

S2) Arima, H., Kasem, M. R. & Mizuguchi, Y. Axis thermal expansion switching in transition-metal zirconides $TrZr_2$ by tuning the *c*/*a* ratio. Preprint at https://doi.org/10.48550/arXiv.2210.10367 (2022).
